# A new species of *Cheiloneurus* Westwood (Hymenoptera, Encyrtidae) as a hyperparasitoid of the invasive cotton mealybug, *Phenacoccus solenopsis* Tinsley, in China

**DOI:** 10.3897/zookeys.974.55528

**Published:** 2020-10-07

**Authors:** Zhuomiao Li, Tingting Yao, Zhihong Xu, Ling Meng, Baoping Li

**Affiliations:** 1 School of Plant Protection, Nanjing Agricultural University, No. 1 Weigang, Nanjing, Jiangsu 210095, China Nanjing Agricultural University Nanjing China; 2 Department of Plant Protection, School of Agriculture and Food Science, Zhejiang Agriculture & Forestry University, Lin’an, Zhejiang 311300, China Zhejiang Agriculture & Forestry University Lin’an China

**Keywords:** *Aenasius arizonensis*, *
bambawalei*, biological control, hyperparasitism, Nanjing

## Abstract

A new species, *Cheiloneurus nankingensis***sp. nov.**, from Eastern China is described. It is similar to *C. arabiacus* Hayat but distinct from it in a number of morphological characters. It is a hyperparasitoid with the encyrtid wasp *Aenasius arizonensis* Girault, 1915 as the primary host and the cotton mealybug *Phenacoccus solenopsis* Tinsley, 1898 (Hemiptera: Pseudococcidae) as the secondary host. A key to all seven species of *Cheiloneurus* known from China is presented.

## Introduction

Hyperparasitoids are specialized natural enemies of primary parasitoids but their negative effects on biological control have often been overlooked. Hyperparasitoids may directly impact their primary parasitoid by parasitizing their offspring and host-feeding (Sullivan 1987; Godfray 1994). Besides, the presence of hyperparasitoids may cause dispersal and patch leaving of their primary parasitoids to further influence biological control efficacy (Cusumano et al. 2019). Hyperparasitoids are often mistakenly regarded as primary parasitoids when they are collected from reared herbivorous hosts that have been parasitized. For example, while *Cheiloneurus* (Encyrtidae) has been recorded as a hyperparasitoid of the cotton mealybug *Phenacoccus solenopsis* Tinsley, 1898 (Hemiptera, Pseudococcidae) in North America (Fuchs et al. 1991), it was considered as a primary parasitoid of the mealybug in China (Li et al. 2020).

*Phenacoccus solenopsis*, native to North America, has spread to Asia over the past two decades (Tong et al. 2018). Since entering China, it has spread over a wide area and caused serious concern over its damage to a variety of economic crop plants (Wang et al. 2020). Biological control with natural enemies provides an alternative approach to managing this invasive pest. Numerous species of parasitoid wasps have been known attacking *P. solenopsis* in invaded Asia countries (Hayat 2009; Chen et al. 2011; Li et al. 2020). Yet few hyperparasitoids of the mealybug have been discovered in these areas.

Here, a hyperparasitoid wasp new to science is named *Cheiloneurus nankingensis* sp. nov. It was collected from rearing mummified *P. solenopsis* mealybugs on okra *Abelmoschus esculentus* (Linn.) Moench. Laboratory observation showed that *C. nankingensis* adults produce offspring when attacking the mealybugs that had been parasitized by *Aenasius arizonensis* Girault, 1915, but failed to do so when confined with healthy mealybugs, indicating that *A. arizonensis* is the host. This host parasitoid, having been synonymized with *A. bambawalei* Hayat, 2009, belongs to the subfamily Tetracneminae in Encyrtidae (Fallahzadeh et al. 2014). It was originally recorded in the USA and is now widespread in Asia where it is generally considered to be a potential biological control agent of the invasive cotton mealybug (Chen et al. 2011; Fallahzadeh et al. 2014; Aga et al. 2016; Li et al. 2020). Yet, its potential may be compromised by hyperparasitoids.

*Cheiloneurus* includes more than 140 species over the world, and all species, for which their biologies are known, are hyperparasitoids, attacking a wide range of parasitoid wasp taxa (Trjapitzin and Zuparko 2004). Six species of *Cheiloneurus* have been recorded from across mainland China, mostly with mealybugs as their secondary hosts; their primary hosts are unknown, except for *C. claviger* Thomson, 1876, which parasitizes *Microterys* encyrtid wasps (Xu and Huang 2004; Mita et al. 2016).

## Materials and methods

Sample individuals of the cotton mealybug were collected from okra plants in a vegetable field and then maintained on potato *Solanum tuberosum* L. seedlings in an insectary. Parasitized mealybug mummies were individually placed in glass vials in which a cotton ball soaked with a 10% honey solution was provided as supplementary food. *Cheiloneurus* adults were mounted on slides following the methods described by Noyes (1982) for identification under a stereoscope (Nikon SMZ25, with NIS-Elements BR software for taking measurements and Zerene Stacker for processing photographs).

The terminology follows Noyes and Hayat (1984).

The following abbreviations are used in the description: POL, distance between the posterior ocelli; OOL, distance between a posterior ocellus and the corresponding eye margin; F1, F2, …, F6, first through sixth funicle segments; NAU, Nanjing Agricultural University, Nanjing, China.

## Taxonomy

### Cheiloneurus nankingensis

Taxon classificationAnimaliaHymenopteraEncyrtidae

Li & Xu sp. nov.

861398F4-3CC0-5BCA-A48E-BED3DE03D118

http://zoobank.org/0A0CAA90-9BAD-4A04-8E4A-64851CE36387

**Figure 1.**
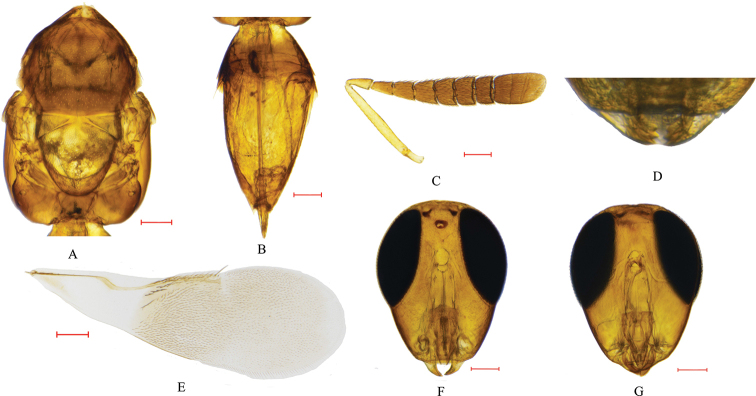
*Cheiloneurus nankingensis* sp. nov. (female, holotype) **A** mesosoma, dorsal view **B** metasoma, dorsal view **C** antennae **D** mandibles **E** fore wing **F** head, front view **G** head, ventral view. Scale bars: 0.10 mm.

**Figure 2.**
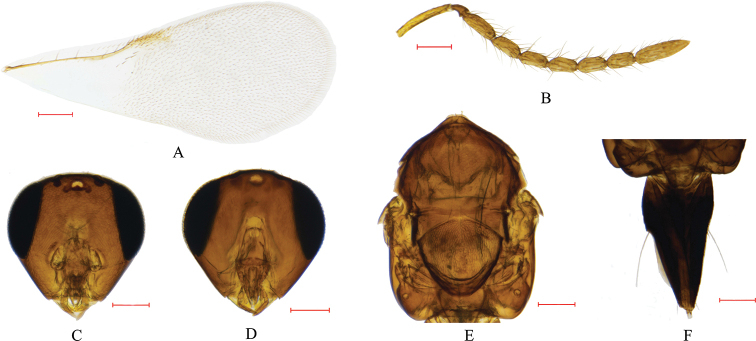
*Cheiloneurus nankingensis* sp. nov. (male) **A** fore wing **B** antennae **C** head, front view **D** head, ventral view **E** mesosoma, dorsal view **F** metasoma, dorsal view. Scale bars: 0.10 mm.

Fig. 1 [female], Fig. 2 [male]

#### Type materials.

***Holotype*.** ♀; China, Jiangsu province: Nanjing city, NAU-affiliated Pailou Experiment station; alt. 18 m; 32°01'10"N, 118°51'21"E; October 2019; Zhuomiao Li leg. ***Paratypes*.** ♀; 15 individuals, same data as for holotype.

#### Deposition.

The type specimens are deposited in the Department of Plant Protection, School of Agriculture and Food Science, Zhejiang Agri & Forest University, Li-an district, Hangzhou city, Zhejiang, China.

#### Etymology.

The species name is derived from the name of the city, Nanjing, where the holotype was collected.

#### Distribution.

All specimens were collected in an eastern suburb of Nanjing city, Jiangsu province, East China.

#### Description.

***Female*.** Holotype, body length 1.54 mm; other females ranged from 1.1 to 1.8 mm. Head dark brown, with purple sheen; ocelli reddish brown; compound eye dark brown; scape, pedicel yellowish brown; funicle and clava black, except first funicle segment with narrow strip of yellowish brown on ventral margin; pronotum and mesoscutum black with yellowish green sheen and silvery white setae; tegulae, axillae, scutellum yellowish brown except middle third of scutellum yellowish white; ventral and lateral sides of thorax dark brown with dark purple sheen; propodeum dark brown. Fore wing infuscated, with basal third, apex narrowly and rectangular area behind stigmal vein, hyaline; hyaline area in basal third with oblique infuscate streak; veins brown; hind wing hyaline. Fore and mid legs yellowish brown except middle part of mid tibia brown; hind femora and tibia brown except tibial base white.

Head: in dorsal view, 0.77 × as broad as high; head width 3.29 × frontovertex width; occipital margin rounded; ocelli arranged in equilateral triangle; POL and OOL 2.02 × and 0.30 × as long as diameter of anterior ocellus respectively; anterior ocellus separated with posterior ocelli by distance 1.01 × as long as POL; frontovertex with scaly reticulations. In frontal view, 0.79 × as broad as high; toruli separated by 1.93 × their own longest diameters; upper margin below lowest level of compound eye; toruli separated from clypeus by distance 0.73 × as long as longest diameter of torulus; mandible tridentate, teeth acute; maxillary palpi with four segments with rounded apex.

Antennae: scape cylindrical, 8.27 × as long as broad; pedicel 2.23 × as long as wide and 1.26 × as long as F1. F6 shortest segment; F1 and F2 longer than wide, the ratio of length to width 1.60 and 1.13, respectively; F3–F6 wider than long, the ratio of length to width 0.87, 0.67, 0.67, 0.64, respectively; club 1.78 × as long as broad, slightly shorter than preceding three funicle segments combined, clava with second suture slightly oblique.

Mesosoma: mesoscutum with striated or scaly reticulations and usually with distinct silvery white setae; notauli virtually absent; axillae and scutellum flat and usually with sculpture scaly; apex of scutellum with a tuft of bristles (bristles are easily lost at making slide but their swelling bases are recognizable); hind margin of scutellum reaching base of propodeum; mesopleura smooth, reaching base of abdomen; propodeum smooth.

Fore wings: 3.01 × as long as broad, with uniform cilia except basal third; submarginal vein with about five setae; submarginal vein 3.90 × as long as stigmal vein; linea calva closed anteriorly by two or three lines of setae and posteriorly closed by 11 or 12 lines of hyaline setae.

Legs: a row of spines at apex of mid tibia; tibial spur 0.96 × as long asbasitarsus ; basitarsus longer than tarsal segments 2–4.

Metasoma: oblong in dorsal view. Ovipositor sheaths yellow. Exserted part of ovipositor sheaths (from slide) 0.15 × gaster length.

Measurements (from slide): length of mid tibia 0.65 mm, mesosoma 0.73 mm, metasoma 0.70 mm, forewing 1.25 mm.

***Male*.** Length 1.07 mm. Body black. Antennae slender with radicle, scape and pedicel dark yellowish brown; funicle and clava black. Oval compound eye and ocelli black. Legs yellowish brown.

Head: in frontal view, approximately triangular with scaly reticulations, 0.97 × as broad as high; POL and OOL 3.00 × and 0.92 × as long as diameter of anterior ocellus, respectively; toruli separated by 0.83 × their own longest diameters; mandible bidentate; toruli diameters 0.69 × and 2.26 × as long as POL and OOL, respectively; distance between compound eyes 3.45 × as long as that between toruli.

Antennae: scape cylindrical, 6.53 × and 0.46 × as long as broad and head height, respectively; pedicel triangular; clava unsegmented, longer than F5–F6 combined; scape 3.35, 1.62, 1.95, 2.13, 2.14, 2.15, 2.18, and 1.05 times as long as pedicel, F1–F6, and clava, respectively; F1 slightly longer than F2–F6 separately; F2–F6 nearly same length; ratio of length to width 2.57, 2.20, 2.30, 2.22, 2.15, and 2.18 for F1 to F6, respectively.

Mesosoma: In dorsal view, 1.35 × as long as width; mesoscutum, axillae, and scutellum with sculpture scaly and similar to head sculpture; scutellum 0.97 × as long as height; setae and notauli virtually absent.

Fore wings: 2.42 × as long as broad; submarginal vein with about 13 setae; postmarginal and submarginal veins 0.58 × and 10.65 × as long as stigmal vein.

Legs: mid tibia with row of spines apically; spur 1.08 × as long as basitarsus.

Metanotum: nearly triangular in dorsal view. Shorter than thorax.

Measurements (from slide): mesosoma length 0.55 mm, metasoma length 0.43 mm, antennae 0.86 mm, and mid tibia length 0.43 mm.

#### Diagnosis.

This new species resembles *C. arabiacus* Hayat, (2014) but differs from it in the following respects (compared with *C. arabiacus* in brackets): without an infuscate area below proximal half of parastigma (with it); scape cylindrical (slightly expanded in the middle); pedicel as long as F1 (longer than F1–F2 combined); F3 quadrate or slightly broader than long (F3 longer than broad); F4–F6 broader than long (F4, F5 quadrate); clava shorter than F4–F6 combined (longer than F4–F6 combined); head in frontal view higher than broad (broader than high); mandible with three acute teeth (with two acute and one round teeth).

#### Biology.

Little has been known about biology of this new species. It is a hyperparasitoid with the encyrtid wasp *A. arizonensis* as the host, which is a primary and solitary parasitoid of the cotton mealybug *P. solenopsis*. This hyperparasitoid attacks only mealybugs that have already been parasitized by *A. arizonensis* and the number of offspring hyperparasitoids ermerging from a mummified mealybug ranges from one to 18. The prevalence of hyperparasitism by this wasp across the range of the cotton mealybug remains to be investigated.

### Key to females of *Cheiloneurus* species known from China

**Table:** 

1	Scutellum without a tuft of bristles at apex	***C. exitiosus* Perkins, 1906**
–	Scutellum with a tuft of bristles at apex	**2**
2	Scape cylindrical; clava slightly broader than funicle	**3**
–	Scape slightly expanded in the middle; clava clearly broader than funicle	**5**
3	F1–F6 longer than wide, entirely whitish	**4**
–	Only F1–F2 longer than wide; funicle blackish	***C. nankingensis* sp. nov.**
4	Pedicel as long as F1; clava as long as F4–F6 combined	***C. quercus* Mayr, 1876**
–	Pedicel longer than F1; clava as long as F3–F6 combined	***C. sinensis* Özdikmen, 2011**
5	Fore wing apex narrowly hyaline; funicle entirely blackish	***C. axillaris* Hayat, Alam & Agarwal, 1975**
–	Fore wing apex narrowly not hyaline; some funicular segments whitish	**6**
6	F4–F5 whitish with a brown stripe on ventral margin	***C. chinensis* Shi, 1993**
–	F4–F5 entirely whitish	***C. claviger* Thomson, 1876**

## Supplementary Material

XML Treatment for Cheiloneurus nankingensis
